# Non-Lethal Sequential Individual Monitoring of Viremia in Relation to DNA Vaccination in Fish–Example Using a Salmon Alphavirus DNA Vaccine in Atlantic Salmon *Salmo salar*

**DOI:** 10.3390/vaccines9020163

**Published:** 2021-02-17

**Authors:** Catherine Collins, Katherine Lester, Jorge Del-Pozo, Bertrand Collet

**Affiliations:** 1 VIM, UVSQ, INRAE, Université Paris-Saclay, Domaine de Vilvert, 78352 Jouy-En-Josas, France; 2 Marine Scotland, Aberdeen AB11 9DB, UK; Katherine.Lester@sasa.gov.scot; 3 SASA (Science and Advice for Scottish Agriculture), Edinburgh EH12 9FJ, UK; 4 The Royal (Dick) School of Veterinary Studies and The Roslin Institute, Easter Bush Campus, Midlothian, Edinburgh EH25 9RG, UK; Jorge.Del.Pozo@ed.ac.uk

**Keywords:** SAV1, DNA vaccine, Viremia, reporter assay, individual fish monitoring, salmon

## Abstract

Traditionally, commercial testing for vaccine efficacy has relied on the mass infection of vaccinated and unvaccinated animals and the comparison of mortality prevalence and incidence. For some infection models where disease does not cause mortality this approach to testing vaccine efficacy is not useful. Additionally, in fish experimental studies on vaccine efficacy and immune response the norm is that several individuals are lethally sampled at sequential timepoints, and results are extrapolated to represent the kinetics of immune and disease parameters of an individual fish over the entire experimental infection period. In the present study we developed a new approach to vaccine testing for viremic viruses in fish by following the same individuals over the course of a DNA vaccination and experimental infection through repeated blood collection and analyses. Injectable DNA vaccines are particularly efficient against viral disease in fish. To date, two DNA vaccines have been authorised for use in fish farming, one in Canada against Infectious Haemorrhagic Necrotic virus and more recently one in Europe against Salmon Pancreatic Disease virus (SPDv) subtype 3. In the current study we engineered and used an experimental DNA vaccine against SPDv subtype 1. We measured viremia using a reporter cell line system and demonstrated that the viremia phase was completely extinguished following DNA vaccination. Differences in viremia infection kinetics between fish in the placebo group could be related to subsequent antibody levels in the individual fish, with higher antibody levels at terminal sampling in fish showing earlier viremia peaks. The results indicate that sequential non-lethal sampling can highlight associations between infection traits and immune responses measured at asynchronous timepoints and, can provide biological explanations for variation in data. Similar to results observed for the SPDv subtype 3 DNA vaccine, the SPDv subtype 1 DNA vaccine also induced an interferon type 1 response after vaccination and provided high protection against SPDv under laboratory conditions when fish were challenged at 7 weeks post-vaccination.

## 1. Introduction

Salmonid alpha viruses form a distinct group within the viral genus *Alphavirus*, family Togaviridae. They are pathogens of farmed Atlantic salmon (*Salmo salar* L.) (Salmon Pancreas Disease: SPD) and rainbow trout (*Oncorhynchus mykiss*) (Sleeping Disease: SD) in Europe and are responsible for serious losses to the aquaculture industry, through direct fish mortality and loss of condition, but also indirectly through complicating treatments for other diseases [1]. The clinical symptoms consist of loss of appetite and lethargy, with histological signs of pancreatic necrosis, necrotizing myocarditis, and necrotizing skeletal myositis/oesophagitis. Due to damage of the heart and skeletal muscle, handling of fish and associated stress can result in increased mortalities. The disease is normally managed through minimising stressful situations or activities and controlling feeding. Two vaccines, based on inactivated virus, have been developed by Merck Animal Health (MSD) and approved for use against SPD, a monovalent vaccine NORVAX^®^ Compact PD, and a multivalent vaccine including inactivated Salmon Pancreatic Disease Virus (SPDv), AQUAVAC^®^ PD3/PD7, both against SPDv subtype 1. In 2017, a DNA vaccine against SPDv subtype 3, which is the predominant subtype associated with SPD in Norway, was approved for use by the European Medicines Agency and is currently being trialled in commercial salmon farms.

The majority of vaccines used in aquaculture are inactivated non-replicating virus vaccines [2]. Live attenuated virus and DNA vaccines have, however, shown higher levels of viral protection in fish compared to the other formats [3]. This may be due in part to the production and presentation of the antigen in a more natural form. Other advantages of DNA vaccines include the ability to induce both humoral and cell immune responses, often without the requirement for any adjuvant; a potential lower production cost relative to traditional viral vaccines; and the fact that changes in antigen sequence or other elements can be rapidly incorporated [4]. A DNA vaccine, based on the virus glycoprotein, for Infectious Haemorrhagic Necrosis virus (IHNv) has been approved and used in Canada since 2005 with no reports of disease outbreaks in vaccinated fish [3]. In experimental trials, a DNA vaccine for Viral Haemorrhagic Septicaemia virus (VHSv), again based on the virus glycoprotein, was highly effective in protecting against VHS in trout [5]. There are, however, also accounts of poor success in experimental trials for DNA vaccines against other pathogens such as Infectious Pancreatic Necrosis virus (IPNv), Infectious salmon anaemia virus (ISAv) or SPDv [6]. Summaries of DNA vaccine efficacy for different fish viruses have been reviewed previously [3,7]. Development of a successful DNA vaccine is therefore not a forgone conclusion, and differences between viral species, such as tropism, mode of infection and replication, as well as the antigen expressed and differences in vaccine strategy, will influence vaccination outcome.

Infection with a live pathogen (through more natural routes, e.g., bath/co-habiting with shedder fish), and the host immune response following infection/vaccination, is often subject to variation between individuals in amplitude and/or kinetics; therefore, monitoring individual animals is of benefit to obtaining robust data [8] and is routinely used in studies using larger vertebrates. Although more complicated to implement for aquatic vertebrates, this approach has been demonstrated in fish for a number of pathogens, including viral pathogens, through repeated collection of small blood samples [9,10]. This paper describes the development and preliminary testing under laboratory conditions of a non-lethal sampling approach in fish to monitor infection dynamics and vaccine efficacy. Additionally, given the requirement for analysis of small blood volumes from young fish, the utility of a reporter-based system for analyss of viremia and the presence of neutralising antibodies has been demonstrated. The methodology was tested using an experimental DNA vaccine against SPDv subtype 1, found in Scottish and Irish aquaculture sites. The DNA vaccine expresses all SPDv subtype 1 structural proteins. Its efficacy in controlling SPDv subtype 1 in relation to viremia suppression, presence of virus in target tissues, and histopathological damage to tissues under laboratory conditions is presented, alongside data on immune gene expression following preliminary vaccine testing. The latter were also used to determine if relevant immune response at the site of injection could also be detected systemically in blood, as a basis for monitoring response in a non-lethal sequential sampling approach.

## 2. Material and Methods

### 2.1. DNA Vaccine

The DNA vaccine (referred to as ppG) was constructed as described by [11] and consisted of a polyprotein of the SPDv subtype 1 structural genes with a GFP reporter protein inserted at the N-terminal, linked to the viral capsid protein. The plasmid was prepared using Qiagen Endofree Gigaprep according to the manufacturer’s instructions. Prior to injection, the solution was prepared in saline to a final concentration of 1 µg/µL and filtered-sterilised. The control plasmid was the vector used to construct the vaccine (pcDNA3.1-Hyg referred to as 3.1H or empty vector) and was prepared in an identical way to that of the vaccine ppG.

### 2.2. Ethical Statement

Animal experimentation was carried out at Marine Scotland Science, Aberdeen, in accordance with the UK Animals (Scientific Procedures) Act 1986 (ASPA) under the project licence PPL3965. The protocol was validated by a professional statistician and approved by the Marine Scotland Ethical Review Committee. All procedures were performed under MS222 anaesthesia, and all efforts were made to minimise suffering.

### 2.3. Experiment 1—Host Immune Response Following DNA Vaccination (Fish Not Challenged—Lethal Sampling)

One hundred and eight (108) Atlantic salmon *Salmo salar* pre-smolts 49.4 ± 8.2 g (Av. ± SD) were Passive Integrated Transponder (PIT)-tagged (i-Tag 162; Biomarks, Boise, USA) and maintained in freshwater at 13 °C. A health check was performed on five additional fish, with qPCR and culture techniques used to screen for viral and bacterial pathogens, including SPDv. The fish were anaesthetised in 0.08 g/l MS222 (Sigma, Gillingham, UK), weighed and injected intramuscularly (*n* = 36 per group) in two sites (25 µL in each site) with either the empty plasmid, the vaccine ppG or PBS using a 27 G 0.4 × 12 mm needle (Terumo, Myjector 0.5 syringe). The same amount of total plasmid DNA (50 µg i.e., 25µg plasmid per injection site) was injected into fish in the former two groups. Care was applied when withdrawing the needle to avoid leakage of the DNA solution. The site of injection was midway between the lateral line and the posterior edge of the dorsal fin and midway between the lateral line and anterior edge of the adipose fin. Groups were injected in a randomised order, washed in 10 L water per group for 5 min during recovery before being distributed into 6 replicate 350 L tanks (each tank containing 6 fish per group, 18 fish in total).

**Figure 1:**
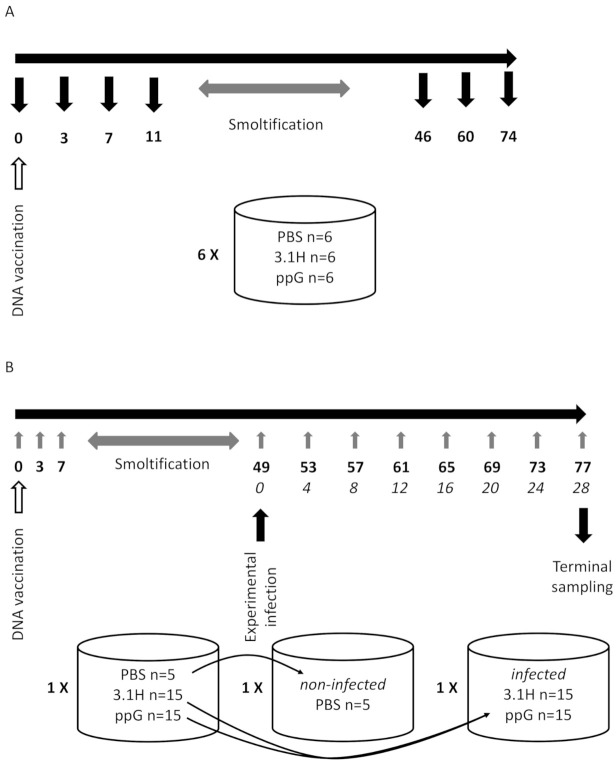
Overview of experimental design for Salmon Pancreatic Disease virus (SPDv) DNA vaccination of salmon. (**A**). Experiment 1 (lethal sampling, vaccination only, no viral challenge). (**B**)**.** Experiment 2 (non-lethal sampling, vaccination followed by immersion challenge). Numbers in bold correspond to days post-vaccination, numbers in italic correspond to days post infection, grey upward arrows correspond to non-lethal blood collections, and black downward arrows correspond to lethal tissue sampling. ppG is the DNA vaccine (pcDNA3.1-Hyg-mEGFP-pp4640 plasmid containing SPDv structural polyprotein), and 3.1H is the empty vector control (pcDNA3.1-mEGFP).

The fish were progressively acclimatised to seawater by increasing salinity by 50% between 3 and 7 days post-vaccination (dpv) and to full seawater between 7 and 11 dpv. At 3, 7, 11, 46, 60, and 70 dpv, six fish per group (one full tank) were sampled. Tissues were sampled including muscle tissue at the site of injection and kidney and stored in 750 µL RNAlater (Sigma, Gillingham, UK) at −80 °C until processed. Blood was withdrawn from the caudal vein and added to tubes containing 10 µL heparin (10 units in PBS; Sigma, Gillingham, UK) and kept on ice. Immediately after collection, the blood samples were centrifuged at 13,000× *g* for 30 s and the plasma and pelleted blood cells stored separately at −80 °C until analysed. An overview of the experimental design is given in Figure 1A.

### 2.4. Experiment 2—Non-Lethal Sampling, Vaccination Followed by Immersion Challenge

#### 2.4.1. Vaccination

Atlantic salmon *Salmo salar* pre-smolts (*n* = 35, stock from AquaGen, Norway) were held in a 350 L tank in a flow-through system, with a flow of 3 L/min. The fish were fed 1.5% body weight per day on 3 mm Nutra advanced 50+ (Skretting, Stavanger, Norway) and held in ambient lighting (Aberdeen, Scotland, September-November). The pre-smolts 63.3 ± 10.4 g (Av. ± SD) were PIT-tagged (i-Tag 162; Biomarks, Boise, USA) and maintained in freshwater as previously at 13 °C for 1 week post-tagging prior to vaccination. A health check was performed as for experiment 1.

Prior to vaccination, the fish were anaesthetised in 0.08 g/L MS222 (Sigma, Gillingham, UK), and a blood sample (50 µL) (day 0) was collected from the caudal vein using a 27 G 0.3 × 8 mm needle (Myjector 0.5 syringe, Terumo) and added to tubes containing 10 µL heparin (10 units in PBS; Sigma, Gillingham, UK) and kept on ice until stored, as described previously. In order to minimise the tissue damage during repeat blood collection, the needle was inserted within the sagittal plane through the skin and conjunctive tissues between the muscle somites to reach the caudal vein. The fish were then injected intramuscularly, again using a 27 G 0.3 × 8 mm needle, with solutions corresponding to 50 µL (25 µL in two sites) of PBS (*n* = 5, group PBS), or pcDNA3.1-Hyg (*n* = 15, group 3.1H/empty vector) or a 1:1 mixture of pcDNA3.1-Hyg and ppG (*n* = 15, group ppG/vaccine). A total amount of 50 µg plasmid in 50 µL volume was injected per fish. The site of injection was on the left side of the fish at the same locations as described above in experiment 1.

PIT tag numbers associated with fish from each group were recorded, and all fish returned to the same single tank at a stocking density of approx. 5 kg/m^3^. A blood sample was collected as described above at 3 and 7 days post-vaccination (dpv), prior to smoltification. During smoltification, the fish were maintained as previously but were subjected to a L:D 12:12 photoperiod. The fish were exposed to progressive increases in salinity to full seawater and completed smoltification between 35 and 41 dpv.

#### 2.4.2. SPDv Challenge

SPDv (subtype 1, strain F07-220, kindly donated by AFBI-Ireland), grown at 15 °C on CHSE-214 cells in culture medium, was harvested and frozen at −80 °C until use. At 49 dpv (0 days post infection-dpi), a 50 µL blood sample was collected from all fish (weight = 69.6 ± 15.1 g, Av. ± SD). The fish from the 3.1H/empty vector group and the ppG/vaccine group were transferred together into a single tank with 40 L aerated water. SPDv was added to the tank to a final titre of 10^4^ TCID_50_/L. The fish were left for 1 h and then transferred into a single 350 L tank. Fish from the PBS group were kept in a separate 350 L tank (uninfected controls). Non-lethal blood samples (50 µL) were collected and processed as described above from all fish at dpv 53, 57, 61, 65, 69, and 73 corresponding to dpi 4, 8, 12, 16, 20, and 24, respectively. Terminal blood and tissue were sampled at dpv 77 (dpi 28), with tissue (heart and muscle) stored in RNAlater (Sigma, Gillingham, UK) for viral load qPCR analysis and in 10% Neutral Buffered Formalin (Cellpath Ltd., Powys, UK) for histopathology analysis. An overview of the experimental design is provided in Figure 1B.

### 2.5. Measurement of Viremia and Neutralising Antibodies by RTG-P1 Assay (Experiment 2)

RTG-P1 cells (*mx1* promoter reporter system) (ATCC CRL 2829 [12]) were seeded in culture medium (L-15 Lonza, Belgium; 10% FBS Sigma, Gillingham, UK) on 96-well plates (Greiner, Gloucestershire, UK) 24 h before use. To measure viremia, individual fish plasma (5 µL into 200 µL culture medium) from all experimental groups, from all sampling points post-infection, was applied to individual wells, in four replicates, randomised across plates, and the cells were incubated for 14 days at 15 °C. RTG-P1 cells in three wells were left unstimulated in each plate as background controls. The luciferase activity at day 14 was measured by draining the culture medium from wells and adding 75 µL of SteadyGlo Luciferase substrate (Promega, UK) to each well. The light emission was measured over 10 s using a Wallac Victor3 1420 Multilabel counter set up for luminescence (Perkin Elmer, Cambridge, UK) as Relative Light Units (RLU). Inducibility for each sample was calculated as described previously [12]; briefly, the RLU values for the stimulated cells (*n* = 4 wells per plasma sample) were divided by the average RLU values in sample wells with no plasma (*n* = 3 per plate) to correct for plate-to-plate variations.

In order to estimate the neutralising ability of plasma samples collected at dpv 49/dpi 0 and the terminal timepoint (dpv 77/dpi 28), equal amounts of virus inoculum (25 µL of a titre of 10^3^ TCID_50_/L) and plasma (25 µL diluted 1/20) was incubated for 1 h at 15 °C. Additionally, plasma (25 µL diluted 1/20) was incubated with media (25 µL) as a control to correct for cytotoxicity or endogenous viremia in the infected fish samples. Each mixture was applied directly to RTG-P1 and left for 1 h to allow any unbound virus to adsorb to cell surface before adding 160 µL medium per well of a 96-well plate (Greiner, Gloucestershire, UK) and incubated for 7 days at 15 °C. The medium was drained from the plate, and 50 µL of SteadyGlo Luciferase substrate (Promega, Southampton, UK) was added per well, light emission was measured, and the Relative Light Units (RLU) was recorded. The neutralisation level was defined as the RLU measured in the wells containing plasma only divided by the luminescence measured in its counterpart well containing the plasma plus standard virus amount. A value of 1 indicates complete neutralization, and values below 1 indicate partial neutralisation. Luciferase inducibility in the RTG-P1 reporter cell line indicates induction of IFN pathways due to presence of virus particles, as levels of IFN itself in plasma has been found to be insufficient to generate a detectable signal with the RTG-P1 reporter assay [12].

### 2.6. SPDv Re-Isolation and Confirmation from Plasma Samples (Experiment 2)

Due to the limiting amount of plasma available after trial tests, it was not possible to attempt re-isolation of virus from each individual fish at every timepoint. Virus isolation was attempted from plasma samples from a total of 9 and 8 fish, respectively, from the 3.1H/empty vector group and ppG vaccinated group at sampling points 4 and 12 dpi. Two fish from the 3.1H/empty vector group were also tested at 16 dpi. The plasma from the challenged fish was diluted serially 10-fold and inoculated onto a cell monolayer of CHSE-214 cells seeded 24–48 h previously in a 96-well plate (Greiner, Gloucestershire, UK). Cultures were incubated at 15 °C and monitored for cytopathic effect (CPE) at days 7 and 14 post-inoculation. To confirm the presence of SPDv, supernatants at day 14 post-inoculation were passed to a fresh cell monolayer and incubated for 6 days at 15 °C, and the cells then fixed with acetone for further testing using an immunofluorescence assay (IFAT). Briefly, fixed cells were incubated with SPDv specific mAbs (17 H23 anti-E2, [13]) followed by a fluorescein-conjugated anti-mouse immunoglobulin (F0257, Sigma, Gillingham, UK). Cultures were then examined with a UV light microscope (EVOS Imaging System, Thermo-Fisher, Loughborough, UK) under × 20 magnification for the presence of staining.

### 2.7. Heart and Muscle Histopathology Scoring (Experiment 2)

Heart and muscle tissue were sampled from each fish, surviving to termination, at 28 dpi (77 dpv). Muscle tissue was excised from the site of vaccine injection (approximately 0.5 cm^3^). Tissues were left in 10% neutral buffered formalin for a minimum of 24 h, routinely processed and paraffin-wax-embedded. Sections were cut at 3 µm and stained with haematoxylin and eosin (H&E). Scoring of histological changes in heart and muscle was performed following the criteria given in [14] (Table 1).

### 2.8. RNA Extraction and Gene Expression (Immune Gene, Virus Load, Plasmid Load; Experiment 1 and/or 2)

In experiment 1, total RNA was purified from head kidney and blood using RNeasy mini kit (Qiagen, Manchester, UK) according to the manufacturer’s recommendations and described in greater detail below. Total RNA and DNA were purified from the same skeletal muscle sample and collected at the site of injection using Allprep mini kit (Qiagen, Manchester, UK) according to the manufacturer’s recommendations. For the tissues collected in experiment 2, the RNA extraction procedure was carried out using the Qiagen RNeasy kit (Qiagen, Manchester, UK) according to the manufacturer’s instructions. The skeletal muscle and heart (half of sagittal dissection of whole heart) or 50 µL total blood cells (from pellet) were homogenised in 1 mL RLT+-BME (RLT+ buffer, Qiagen with 1% *v/v* β-mercaptoethanol, Sigma, Gillingham, UK) using a tissue lyser and a 5 mm stainless steel bead (Qiagen, Manchester, UK) for 3 min at 25 Hz (1 min at 25 Hz for the blood cells) as described previously [9]. Depending on the viscosity of the skeletal muscle tissue homogenate, a volume of 50–100 µL was used in subsequent RNA extraction steps, whereas 300 µL of heart or blood cell homogenate (or kidney homogenate in experiment 1) was uniformly used. RNA was quantified using a nanodrop (Labtech International) and stored at −80 °C until being processed further. RNA from heart and muscle were pooled 1:1. The RNA was reverse-transcribed into cDNA using High-Capacity cDNA Reverse Transcription Kit (Applied Biosystems, Thermofisher scientific, Loughborough, UK) according to the manufacturer’s instructions and the cDNA diluted 1 in 4 with molecular grade water (Sigma, Gillingham, UK). qPCR was carried out using TaqMan probes (Thermoscientific) (at a final concentration of 250 nM for the probe and 900 nM for each primer), 2 × OneTaq mastermix (New England Biolabs, Hitchin, UK) in a 10 µL total volume reaction on a Light Cycler (Roche) using a two-step cycling protocol (45 cycles of 94 °C 15 s, 60 °C 1 min). Assays for Elongation factor α (ELF α), LOC100136525, (used as a normalisation factor for quantity of cDNA in reaction, indication of successful cDNA synthesis, and presence of any inhibitory factors), *mx* (LOC100136920), *cd83* (LOC100136479, LOC106581291), *cd8* (LOC100136450), γ*IP* (LOC100196194), *arginase* (LOC100500786), *il1β* (LOC100136449), *inos* (LOC100136358) genes, and SPDv non-structural protein 1 (nsp1) genes are described previously [15,16,17]. Heart and muscle and blood cDNA from the terminal sampling point of the SPDv challenge experiment (experiment 2) were assayed for virus presence and levels. The expression levels of virus or immune genes were expressed relative to that of host ELF.

The level of plasmid in muscle at the site of injection in experiment 1 was quantified from the purified DNA (Allprep mini kit, Qiagen, Manchester, UK) using the method described previously [18] using a specific TaqMan assay designed against the CMV promoter region of the vector PCMV-F 5′- GTGATGCGGTTTTGGCAGTAC -3′, PCMV-R 5′-TGGAAATCCCCGTGAGTCAAAC-3′, and a PCMV probe 5′-6FAM-ATCCACGCCCATTGAT-3′.

### 2.9. Data and Statistical Analyses

Values obtained for gene expression and neutralising antibody levels were compared between the different experimental groups using a two-tailed t-test and unequal variance. *p*-values were adjusted based on the Benjamini-Hochberg method [19] and a false discovery rate (FDR) of 0.05 to correct for type I errors in significance due to multiple comparisons.

To compare the non-lethal sequential sampling approach to the more traditional lethal sampling approach, an analysis was performed to generate theoretical viremia kinetics from sequentially repeat-sampled infected fish in the 3.1H/empty vector group, which would approximate a lethal sampling regime; i.e., a lethal sampling regime representing viremia measurements taken from individual, different, fish killed and sampled at different time points post-infection, and plotted to represent the overall group viremia kinetics for the infection challenge. Individual fish in the 3.1H/empty vector group were assigned randomised numbers using the randomisation function in Excel, and the values were sorted in ascending order. Three fish were then assigned to a single specific sampling point, and the values obtained for their viremia at that specific time point were used to represent the counterpart “lethal sampling“ value for that time point. These fish (remembering that in the non-lethal method they are sampled at all time points) were then excluded from the further generation of viremia values. As only 13 fish were available from the actual non-lethal experiment (2 having died over the experimental period), to avoid resampling of the same fish due to the dependent nature of the successive samples, only the time points 8 dpi to 20 dpi were used, as these represented the time points where viremia peaks were observed in the non-lethal blood samples. The first three fish were assigned to sampling point dpi 8, the next to dpi 12 and so on until 20 dpi. To “anchor” the simulated graphs visually, the averaged non-lethal viremia value obtained from all 13 fish in the 3.1H/empty vector group was calculated for 0, 4, 24, and 28 dpi, and these averaged values used for all graphs. The above randomisation procedure and calculation of average viremia values for sampling points 8, 12, 16, and 20 dpi was repeated 10 times, and graphs were generated from each set of values.

The individual viremia kinetics based on sequential non-lethal samples were analysed using a specific method described previously by [9].

## 3. Results

### 3.1. Immune Gene Expression Following Vaccination-Experiment 1 (Lethal Sampling)

Results based on unadjusted *p*-values are presented in some cases because the trend supports findings of gene expression at significant levels in previous SPDv DNA vaccine studies (see discussion).

**Figure 2:**
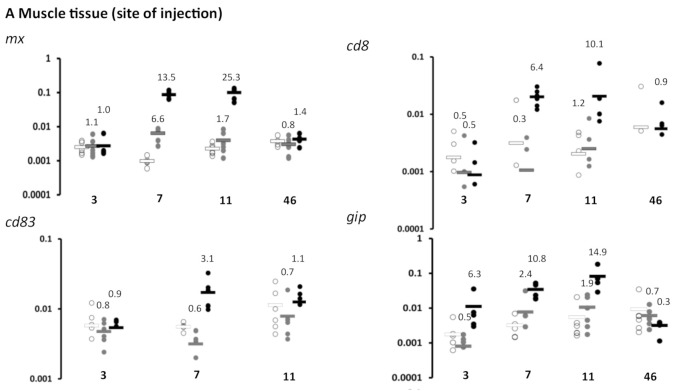

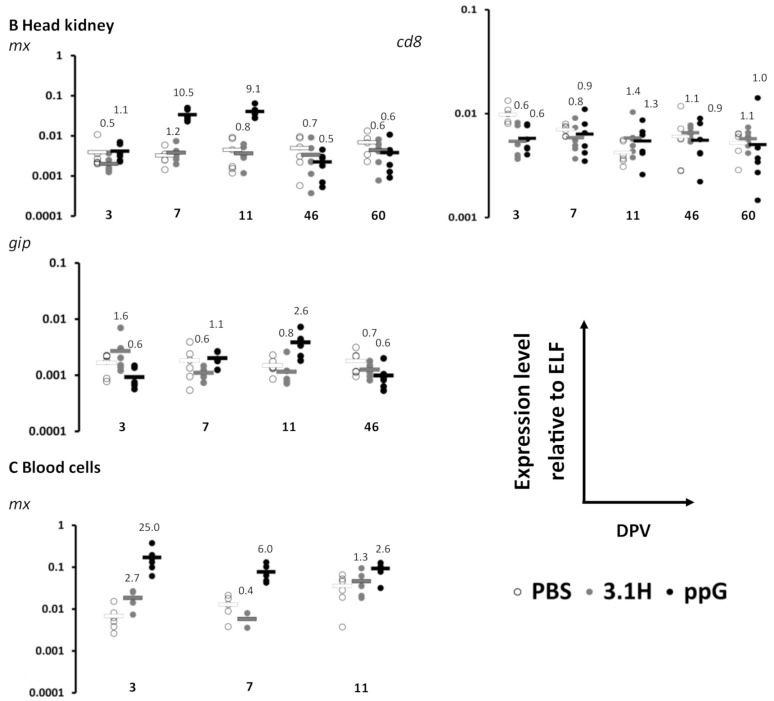
Kinetics of gene expression from the lethal experiment (Exp 1) in animals injected (i.m.) with PBS, vector only (3.1H), or the DNA vaccine (ppG) in the muscle tissue at the site of injection (**A**), in the head kidney (**B**), or in the blood cells (**C**), for *mx*, *gip*, *cd8,* or *cd83*. Individual data are represented as well as the average (horizontal line) (*n* = 6), the time point is indicated as day post-vaccination (dpv), and the fold change in gene expression in ppG and 3.1H groups relative to the PBS group is given.

Once *p*-value thresholds were adjusted for multiple comparisons, of the genes analysed, only the interferon type I (IFN1) induced gene *mx* and the T cell marker gene *cd8* showed significant changes in gene expression, being significantly upregulated in the ppG DNA vaccinated group compared to other groups. *mx* expression was significantly increased in ppG at 7 dpv, and at 7 dpv and 11 dpv, at the site of injection and the kidney, respectively (Figure 2A,B, Table 2), while significant increases in *cd8* expression were observed at 7 dpv at the site of injection (Figure 2A, Table 2). The significant upregulation of gene expression in the ppG group supports a specific host response to nucleotide sequence, expression and/or protein presentation of the polyprotein moiety of the DNA vaccine. A decrease (not significant following *p*-value adjustment) in *cd8* mRNA transcripts was observed in kidney of both DNA-vaccinated (0.8-fold) and empty vector (0.6-fold) groups compared to PBS controls at 3 dpv (Figure 2B, Table 2).

Though not significant following *p*-value adjustment, *mx* mRNA levels in blood cells at 7 and 11 dpi were also found to be higher in ppG DNA-vaccinated fish compared to empty vector (13.3- and 2-fold) and controls (25- and 6-fold) (Figure 2C, Table 2). Unfortunately, processing issues resulted in a loss of RNA from blood cell material at earlier and later time points. The results, however, supported the potential for use of blood to monitor important immune responses in non-lethal samples.

Unadjusted *p*-values also indicated higher levels of *mx* (6.6-fold) mRNA transcripts at the site of injection at 7 dpv in the empty vector injected group compared to PBS controls, indicating that the empty vector itself may have induced some level of IFN1 (Figure 2A, Table 2).

The gamma IP-encoding gene *γip* corresponding to the CXC motif chemokine 10, wrongly annotated as scyb7 Platelet basic protein in the *Salmo salar* genome, is indicative of gamma interferon induction pathways. Though not significant after *p*-value adjustment, *γip* showed increased expression at the site of injection at 7 dpv in the ppG DNA-vaccinated fish compared to PBS control (10.8) and empty vector (4.4-fold) injected fish (Figure 2A, Table 2) and a 14.9-fold increase at dpv 11 compared to the PBS control group. Increased expression of *γip* was also found in the head kidney of ppG vaccinated fish at 7 dpv compared to the empty vector group (1.8-fold) and at 11 dpv compared to both the PBS control and empty vector groups (2.6-fold) (3.3-fold) (Figure 2B, Table 2). Based on unadjusted *p*-values, lower levels (0.6-fold) of *γip* mRNA transcripts were found in the head kidney at 3 dpv in the ppG DNA vaccinated group compared to the PBS control group.

*cd83* expression levels at the site of injection increased (not significant after *p*-value adjustment) at 7 dpv in the ppG group compared to empty vector and PBS control groups (5.4- and 3.1-fold respectively). *cd83* transcript levels were not analysed in the head kidney.

No significant differences between groups were observed in il1b, arginase and inos gene expression (data not shown) after *p*-value adjustment, though a decrease (3.7-fold) in expression was observed pre-*p*-value-adjustment for *il1b* at the site of injection in the ppG vaccinated group compared with PBS controls at 3 dpv.

### 3.2. Efficacy of Sea Water Immersion Challenge and SPDv ppG DNA Vaccination–Experiment 2

During the course of the challenge experiment (experiment 2), one fish each from groups 3.1H/empty vector and ppG died during the seawater transfer period, and a further 2 fish died in group 3.1H, one at 7 dpv and one at 74 dpv/25 dpi.

**Figure 3:**
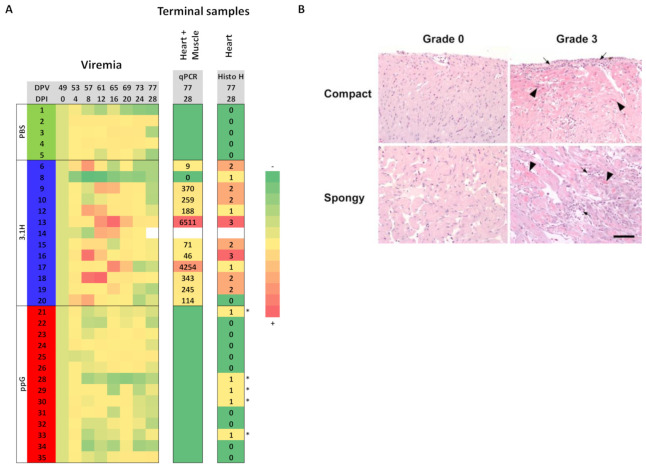
(**A**). Individual SPDv viremia following immersion infection measured by the RTG-P1 method. Fish were injected with PBS (Fish 1–5), 3.1H (empty vector, Fish 6–20) or ppG (SPDv vaccine, Fish 21–30). Data are individual viremia (fish number in the second column) at different days post-infection (DPI) relative to the individual’s viremia at 0 dpi and are represented as a heatmap. The time scale in relation to days post-vaccination (DPV) is also indicated. The column named “qPCR” gives the relative level of viral load assessed by quantitative RT-PCR in a pool of heart and muscle tissue RNA from the terminal sampling point (DPI 28). The column labelled “Histo H” indicates the histopathological score in the heart at dpi 28. “*” indicates a non-inflammatory lesion. (**B**). Compact and spongy myocardium: Histological images of samples from group 3.1H for animals F20 (Grade 0, left) and F13 (Grade 3, right) at terminal sampling date (dpv 77/dpi 28). These samples were chosen to illustrate the differences between the two extremes of the histological presentation (i.e., 0 and 3). Please note that intermediate grades vary in the frequency and severity of the lesions, but not in the specific patterns associated with these lesions. Briefly, the tissue in grade 0 is histologically normal, while the tissue in grade 3 features cardiomyocyte necrosis/degeneration (black arrowheads) and inflammatory infiltration by mononuclear cells (black arrows). These patterns are visible in both the compact and spongy layers. Haematoxylin and Eosin, ×200. Scale bar = 100 µm).

The presence of SPDv was detected by qPCR in pooled heart and muscle tissue in 11 of the 12 (92%) remaining fish at 28 dpi (terminal sampling point) in the 3.1H (empty vector) group (F6-F20) exposed to the virus through bath immersion. The qPCR viral load in group 3.1H, relative to the ELF host housekeeping gene, ranged from 9 (F6) to 6511 (F13) at 28 dpi, with only one negative fish (F8) (Figure 3A). SPDv was not detected by qPCR in pooled heart and muscle tissue in SPDv-exposed fish in the ppG vaccinated group (*n* =14; F21 to F35) at terminal sampling 28 dpi (77 dpv), nor in uninfected PBS injected control fish (*n* = 5; F1 to F5).

No virus was detected by qPCR in any of the blood cell samples from any of the groups at 28 dpi, including the infected 3.1H/empty vector group, indicating that the viremia phase in infected fish had passed and/or was absent.

**Figure 4:**
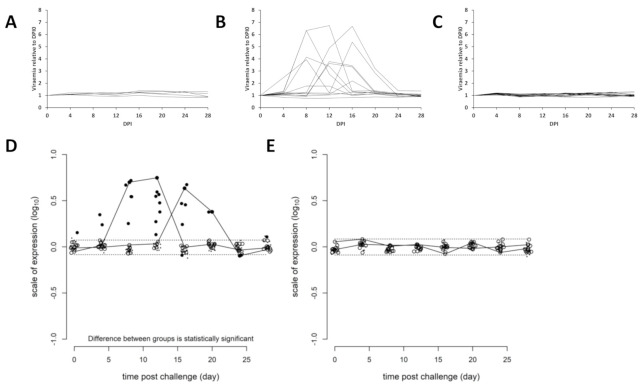
Individual kinetics of viremia in PBS-injected-uninfected (**A**), 3.1H-injected-infected (**B**), and ppG-injected-infected fish (**C**). Statistical analysis using an R-script designed for analysis of individual kinetics (Collet al., 2015) comparing viremia in ppG-injected-infected fish (open circles) and 3.1H-injected-infected groups (close circles) (**D**) and between ppG-injected-infected fish (open circle) and PBS-injected-uninfected groups (close circle) (**E**). The discontinued lines represent the 95% confidence intervals and values outwith these are statistically significant. Two representative individual viremia kinetics are shown in (**D**). 3.3. Viremia Kinetics: Individual Non-Lethal Fish Sampling Versus Simulated Lethal Sampling

Positive viremia, as measured by induction of the RTG-P1 cell line, was detected in 11 of 13 fish, in group 3.1H/empty vector (Figure 4B). Viremia was not detected in group ppG (DNA vaccinated fish) (*n* = 14) nor in uninfected control (PBS injected) fish (*n* = 5) (Figure 3 and Figure 4A,C,E). Overall, the viremia relative to dpi 0 ranged from 0.84 to 1.38, from 0.76 to 6.73 and from 0.85 to 1.31 in the groups PBS-uninfected, 3.1H/empty vector-infected and ppG/vaccine-infected, respectively.

The virus was detected, based on visualisation of IFAT staining in cell cultures exposed to plasma from 2 of the 9 fish tested from the infected 3.1H group (empty vector) at day 4, from 3 of the 9 fish tested at day 12 post-challenge, and from 2 of 2 fish at day 16. The virus was not detected at any stage using plasma from the ppG-vaccinated fish (*n* = 8 at dpi 4 and 12). The results support findings from the RTG-P1 viremia assay, where the virus was not detected in the plasma of ppG vaccinated fish and support the findings of qPCR and histopathological analysis of heart and muscle tissue from the different experimental groups.

Of the 12 infected fish analysed in group 3.1H/empty vector, cardiac lesions were observed in 11, consisting of focal myocardial degeneration (necrosis) and inflammation with scores of 1 (*n* = 3), 2 (*n* = 6) and 3 (*n* = 2). Histopathological features of repair were absent in the heart and skeletal muscle of all samples examined (Figure 3), in line with the time of sampling post-infection i.e., 28 dpi. Histological analysis of heart sections did not detect myocardial degeneration nor inflammation in uninfected control fish (*n* = 5). In ppG-vaccinated infected fish, 9 of 14 fish analysed had a score of 0, and 5 had a score of 1 with respect to cardiac lesions (Table 1, Figure 3A). The cardiac lesions consisted of low levels of myocardial degeneration devoid of inflammation (Figure 3B). The skeletal muscle in all fish analysed had a score of 0 except F30 (ppG group), which had a score of 1 due to a single myofibre degeneration without signs of inflammation (this is interpreted as an incidental finding).

The results indicate that the seawater immersion challenge was successful in transmitting SPDv to Atlantic salmon post-smolts and in inducing SPD. The results also indicate that, within the timescale of vaccination and viral challenge used here, the ppG DNA vaccine for SDPv was highly effective at suppressing viremia, eliminating the virus and preventing disease pathology.

The viremia kinetics are represented separately for the groups PBS/uninfected (Figure 4A), 3.1H/infected (Figure 4B) and ppG/infected (Figure 4C). The kinetics were significantly different in the 3.1H/infected group when compared to the ppG/infected group (Figure 4D), and no significant differences were found in the viremia kinetics between the ppG/infected and PBS/uninfected groups (Figure 4E).

Non-lethal sampling of individual fish over the course of infection demonstrated the presence of two groups of fish within the infected 3.1H/empty vector group with respect to viremia development. One group (*n* = 6) demonstrated the presence of virus in samples taken between days 4, 8, and 12, and the second group (*n* = 5) between days 12, 16, and 20 (Figure 4B,D). Viremia/viremia peak was not observed for two fish.

**Figure 5:**
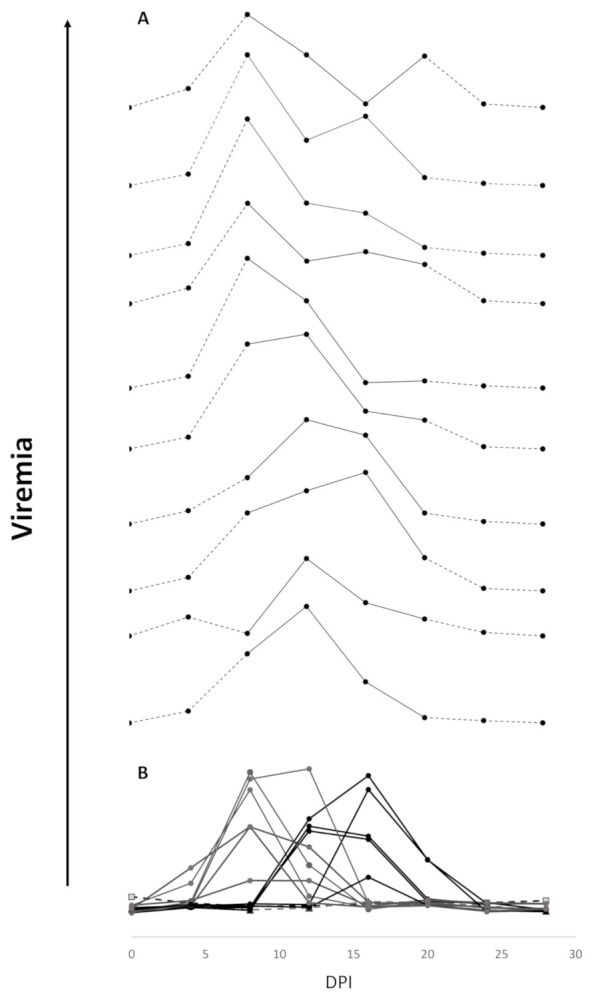
(**A**) Ten theoretical viremia kinetics constructed by randomly resampling individual non-lethal data to model a lethally sampled dataset (*n* = average of viremia levels from 3 different fish for each dpi 7–20). (**B**) Viremia kinetics from actual non-lethal sequentially sampled fish (*n* = 13).

Of the 10 graphs generated from independent samples to simulate viremia kinetics based on lethal individual sampling over 8–20 dpi, eight displayed a single peak, while only two indicated a double peak in viremia within the 3.1H fish group as a whole (Figure 5), the latter reflecting the actual group dynamics based on the non-lethal sampling (though unable to determine a single early or late viremia peak in an individual versus double viremia peaks in the same individual).

### 3.3. Viremia and Mx Gene Expression in Blood Cells–Experiment 2

**Figure 6:**
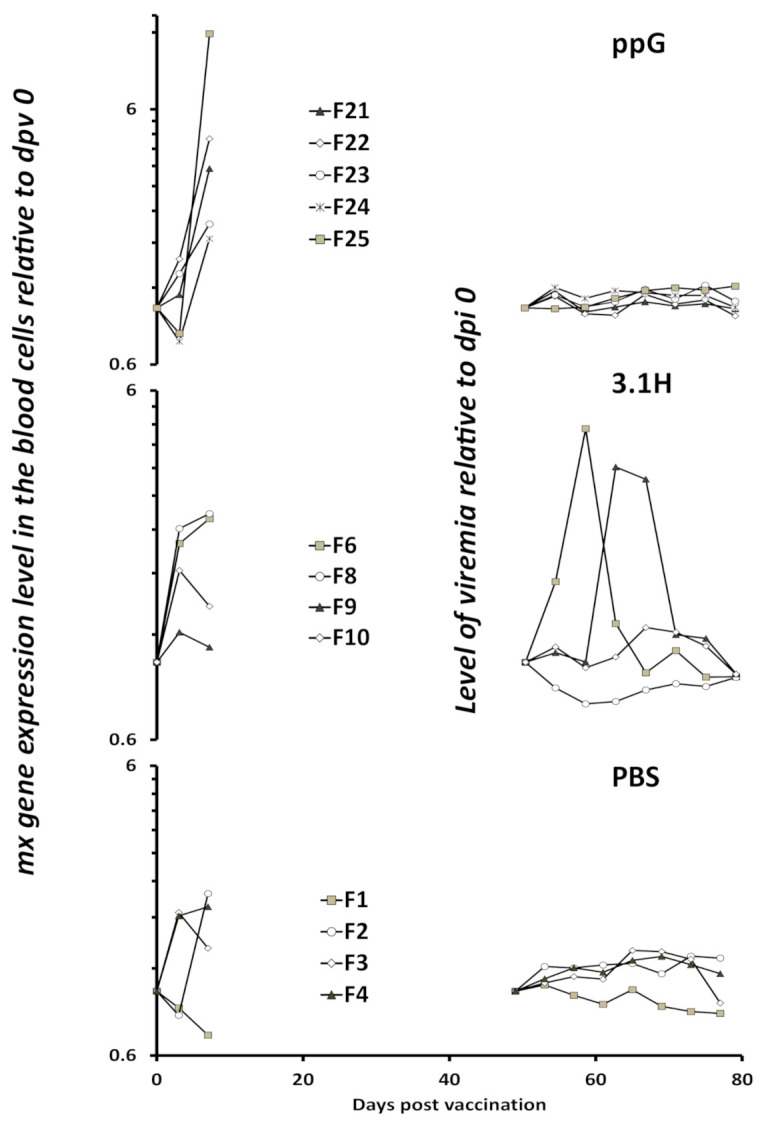
Individual kinetics of *mx* gene expression in blood cells at 3 and 7 dpv and corresponding viremia at dpv 49–77/dpi 0–28 (experiment 2) in the PBS-injected-uninfected group (F1, F2, F3, F4; bottom), 3.1H-injected-infected group (F6, F8, F9, F10; middle) and ppG-injected-infected group (F21, F22, F23, F24, F25; top). The pre-infection data are the relative level of *mx* gene expression in the blood cells expressed as fold change to dpv 0 (pre-vaccination) measured by qPCR (left side, dpv 3 and 7). The post-infection data are the viremia measured in the plasma by the RTG-P1 reporter assay and expressed as fold change to the pre-infected level (dpv 49/dpi 0). The *Y*-axis scale is logarithmic and is identical between the three groups, which have been separated to allow for visualisation of individual kinetics. Minor marker labels indicate an increment of 0.6 (0.6, 1.2, 1.8, 2.4, 3.0, 3.6, 4.2, 4.8, 5.4, and 6.0). Within each group, individuals are uniquely identified by a distinctive marker.

The individual kinetics of both *mx* gene expression in blood cells pre-infection and viremia post-infection are represented in Figure 6. The blood cells from some individuals (5 in group PBS, 4 in group 3.1H and 5 in group ppG) were analysed for *mx* gene expression at 0, 3, and 7 dpv. In the PBS or 3.1H/empty vector groups, the relative level of *mx* gene expression did not go over a value of 2.7, whereas it reached values of 11 in the ppG group. In addition, the average fold changes in the *mx* gene expression level between day 3 and 7 were 1.3, 1.0, and 5.0 in groups PBS, 3.1H, and ppG, respectively. This suggests that the *mx* gene expression level increased only in the ppG-vaccinated group. Significant increases in *mx* expression were also only found in the ppG group following *p*-value adjustment in the analysis of lethally sampled tissue in experiment 1 (Figure 2C).

### 3.4. Presence of SPDv Plasma Neutralising Antibodies—Experiment 2

**Figure 7:**
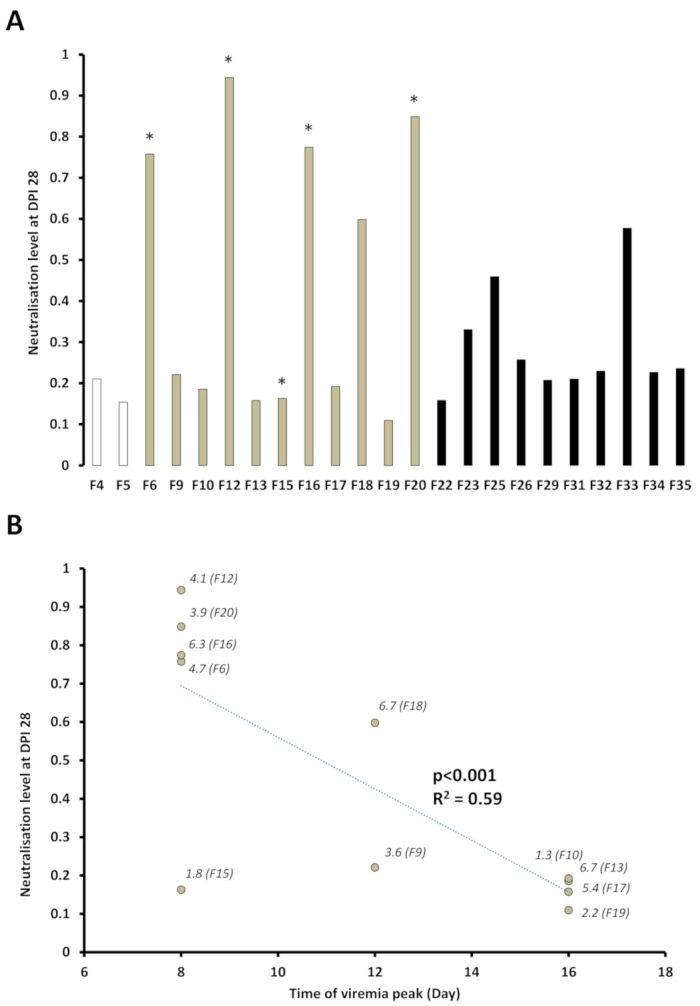
Neutralising activity of the plasma collected at 28 days post-infection (DPI). (**A**) Neutralising antibody levels are indicatedfor. groups: PBS-injected-uninfected group (F4-5, white), 3.1H-injected-infected group (F6-20, grey) and ppG-injected-infected group (F22-35, black). *: animal displaying early peak in viremia (**B**) Correlation between the level of neutralisation at dpi 28 and day (dpi) of viremia peak. The amplitude of the observed viremia peak is indicated on the plot alongside individual fish number in brackets (*F*). The linear regression is indicated by the dotted line with probability and R squared value.

Unfortunately, due to experimental issues with the assay, the kinetics of neutralising antibody responses over the whole experimental period were not obtained for individual fish. When the results were adjusted for multiple comparisons, no significant differences in neutralising antibody levels were observed between empty vector group 3.1H and ppG DNA vaccinated fish at dpv 49/dpi 0, though levels were slightly higher (1.3 fold) in the ppG-vaccinated group. Similarly, no significant differences (adjusted and non-adjusted p-values) were seen between ppG dpi 0 and ppG dpi 28, between 3.1H dpi 0 and 3.1H dpi 28, or between ppG dpi 28 and 3.1H dpi 28 in relation to neutralising antibody levels (Figure 7A). However, based on unadjusted *p*-values, when the 3.1H group was divided into early and late viremia groups, a significant increase in neutralising antibody levels was seen in 3.1H early viremia fish at dpi 28 compared to dpi 0, and significantly higher and lower average antibody levels, respectively, in plasma at dpi 28 in 3.1H early viremia and late viremia groups compared to ppG at dpi 28. A negative trend was observed between the level of neutralisation measured at the end of the infection (dpi 28) and the time of the peak of viremia (Figure 7B).

### 3.5. Kinetics of Plasmid Amount at the Site of Injection (Experiment 1)

**Figure 8:**
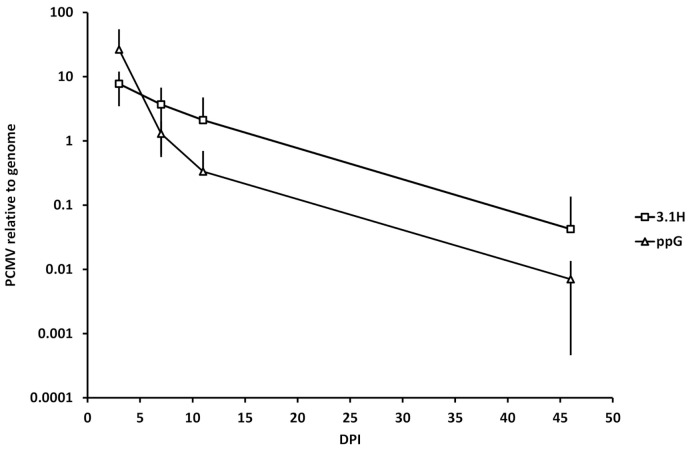
Comparison of the kinetics of the relative amount of plasmid at the site of injection in 3.1H and ppG-injected groups in experiment 1. Data represent average ± standard deviation (*n* = 6) of level of plasmid relative to the host genomic DNA. The scale is logarithmic. PCMV: CMV promoter, present in plasmid).

The level of plasmid present in the muscle at the site of injection from samples collected during experiment 1 was quantified, by qPCR, relative to the level of the host genome for groups 3.1H and ppG at days 3, 7, 11, and 46 after vaccination. Between dpv 3 and dpv 11, the speed of decay was higher for the group ppG (26.5 to 0.3; 98.7% decrease) when compared to the 3.1H group (7.7 to 2.1; 72.8% decrease) (Figure 8).

## 4. Discussion

We have designed an experimental DNA vaccine against SPD in Atlantic salmon using the full SPDv subtype 1 structural polyprotein and demonstrated that, under experimental conditions, viremia was prevented in vaccinated fish following an immersion challenge with the virus at 7 weeks post-vaccination. Virus presence was not detected in heart and muscle tissue, and clinical signs of the disease were additionally absent in vaccinated fish at terminal sampling 4 weeks post-infection. DNA vaccines based on the full structural polyprotein conferring high protection against SPDv infection have previously been reported for SPDv subtype 3 [20] and represent the first approved DNA vaccine (ClyNav, Elanco) for fish use in Europe. Results of vaccine efficacy and data on vaccine-induced immune response presented here for subtype 1 support for the most part published findings for SPDv subtype 3 DNA vaccines.

The current study moreover highlights the advantages of non-lethal sequential blood sampling of fish to define correlates of protection. The measurement of viraemia and antibody neutralisation was obtained from minute amounts of plasma using a reporter cell line RTG-P1 [12], which allowed for individual animals to be repeatedly sampled over the course of infection. This methodology revealed the large variability between individuals in infection dynamics and illustrates for the first time the advantages of monitoring immune parameters individually in fish in relation to vaccine efficacy testing and in relation to interpretation of infection kinetics and immune findings.

Traditional experimental vaccine efficacy studies in Atlantic salmon with the same viral pathogen model (SPDv), and based on lethal sampling, used 35–80 fish per group (35 in [21], 45 in [20], 50 in [22], 60 in [23], 80 in [24]), while studies on viremia kinetics and on neutralising antibodies have used 40–75 fish per group [25,26], though admittedly this also allowed for tissue processing for histopathological data at each time point. The statistical robustness of sequential individual data [8] offers the possibility to reduce the number of animals required, thus addressing 3R aims, and to better interpret the variability in different measures between individuals, which is not possible using the traditional destructive sampling approach. In the present study less than fifteen individuals per group were used to obtain viremia kinetics. The use of an individual sequential monitoring approach has been limited in fish disease and vaccine investigations to date. It has been used previously in salmonid fish by some of the current authors to measure gene expression in blood cells collected repeatedly from the same individuals [9,10,27,28].

### 4.1. Viremia as a Proxy for Vaccine Efficacy

The absence of viremia in the individual fish over the entire challenge period was associated with a lack of clinical signs and absence of virus in target organs at terminal sampling. Viremia therefore acted as a proxy for vaccine efficacy under the conditions in this study, as opposed to traditional approaches, where the animals must be left to develop clinical signs that can be assessed through post-mortem histopathological examination, a time-consuming procedure. However, a more precise correlation analysis remains to be conducted with sub-optimal vaccines to evaluate the sensitivity of the viremia method in assessing vaccine efficacy. The use of a reduction in viremia as a proxy for vaccine efficacy combined with individual monitoring has been used in the past in mammalian models [29,30,31,32] but never in fish, where correlates of vaccine protection would be desirable [33].

### 4.2. Viremia Kinetics

The methodology employed allowed the full viremia kinetics within infected individual fish in the 3.1H empty vector group to be described. Viremia had appeared in some fish by day 3, the first sampling point, and peak viremia was observed between 4–6 days later. Overall, every individual had a single-peak infection kinetic pattern, and overlapping “early” or “late” viremia individuals could be distinguished with approximately 1 week between their viremia peaks. Within the early or late viremia groups, viremia was detected over approximately a 12–16 day period for each group. The results resemble to some degree those obtained by other studies. Desvignes et al. [34] reported virus in serum at 2 days post i.p. injection of SPDv subtype 1 with a maximum average titre by 4 dpi and loss of viremia in some fish by 16 dpi. Christie et al. [35] found viremia in the serum of 50% of fish 4 dpi (i.p.) (though in 90% of fish using RT-PCR), and absence of viremia in all fish at 21 dpi. Similarly, viremia-positive fish numbers had decreased by 21 dpi in i.p. and bath challenges in studies [25,26]. None of these authors identified the existence of fish subgroups with distinct viremia kinetics.

The “late” individuals may either have been more resistant to the cell-culture-derived SPDv subtype 1 isolate used in the bath infection, resulting in a slower initial development of disease, or, more likely, were infected for the first time with virus shed from early viremia individuals. There is a similarity in the lag interval between initial bath exposure and infection in the early viremia group and the subsequent interval between peak viremia in the early group and the first detection of viremia in the late viremia group. There is also similarity in amplitude and duration of early and late viremia kinetics. Though the simultaneous exposure by bath immersion and the numbers of experimental fish may have precluded investigation of an R_0_ reproductive rate for PD/SPDv subtype 1, the individual viral kinetics provide more robust data for certain elements of the R_0_ formula such as the transmission probability per contact and the duration of the infectious period.

The differences in kinetics patterns can be related to waves of infection occurring in experimental fish as suggested, but not explicitly shown, by [14] (for certain SPDv subtypes) and by [26]. These authors found that virus shed in faeces and mucus and/or detected in tank water showed cycles of increases and decreases over the challenge period. There was some suggestion that this pattern may reflect that seen in natural outbreaks [26] but intervals between virus/antibody manifestation in these natural cycles appear much longer [36,37], and likely reflect effects of other environmental or biological parameters. It might, however, be possible that a continuous sequential passing of virus from a low number of fish to others during a viremia stage may maintain the virus within a population until stressful conditions provoke an outbreak, thus contributing to regular “cycles” not dependent on coinciding external virus reservoirs. In terms of experimental studies, references [14,35] found an absence of viremia at later sampling points, thus not supporting a continuous infection. In the latter study, however, infection was standardised by i.p. and resulted in 90–100% prevalence of viremia by 7 and 14 dpi, and the appearance of neutralising antibodies at 21 dpi, at which stage no further viremia was detected. Graham et al. [38] also suggested that SPDv persisted long-term in farmed fish, though this was related to virus in tissues rather than in potential contagious form in blood. Several authors have discussed that SPDv virus persisting in tissues such as heart may contribute to re-infection, but this is not yet shown empirically [35]. Fish in the early viremia group did not become re-infected over the experimental period despite a likely second shedding event from the late viremia group, perhaps due to the development of neutralising antibodies, which have been shown by a number of studies to confer protection against SPDv [39,40]. Nevertheless, if the viral spread is slow in a population, then a large proportion of the fish may remain naïve. Alternatively, antibody titres may decrease over time [35], and it is not clear if a secondary response is present.

### 4.3. Plasma Neutralisation Levels

We used the reporter cell line RTG-P1 to estimate the level of neutralisation activity in the plasma collected from individuals just before viral challenge, corresponding to 49 days post-vaccination (dpv) and, at the end of the infection. Reporter systems have been developed for this purpose in mammalian host/pathogen models [41] but these systems were based on viral-specific promoters, targeted by the virus itself. In the RTG-P1 assay, the luciferase readout is an indirect measure of the viral titre [12] and can be applied to a number of fish viruses capable of replicating in an RTG-derived cell line and activating interferon pathways. Reduction in viral luciferase induction corresponds to neutralisation ability in plasma. When comparing empty vector and ppG vaccine groups at day 49 dpv/0 dpi, no significant difference in neutralising ability was found, though there was an overall trend for slightly higher viral neutralisation in the vaccine group, possibly indicating that the ppG vaccine did not induce a strong response in terms of humoral neutralising antibodies. This is in contrast to the results of [20], who found significantly higher antibody levels in SPDv subtype 3 structural protein DNA-vaccinated fish at 10 weeks post-vaccination, and who postulated that this might have contributed to observed protection following subsequent challenge. Neutralising antibodies are thought to play an important role in alphavirus protection [39,40,42]. The differences seen between the two studies may be due to sampling timing, being 7- and 10-weeks post-vaccination in the current study and in [20], respectively. Neutralising antibodies following SPDv infection are reported to appear by week 2 and reach significantly higher levels by week 6–9 [11,25,43], suggesting that a noticeable antibody response might be expected by 7 weeks post-vaccination if present. However, a previous study by [44] indicated that timing of antibody production post-vaccination (with ISAv DNA vaccine) may be influenced by the *ifn*1paralogue expressed during vaccination, delaying a neutralising response from 7 to 10 weeks, dependent on ifn1 type. We have not determined the *ifn* paralogue(s) expressed in the current study (presence detected based on induction of *mx* gene expression only). The second difference between our ppG vaccine and that of [20] is that ppG has a GFP attached to the capsid protein. This may have prevented the formation and secretion of viral-like particles (VLPs), which could in turn affect antigen presentation via the MHC II pathway and B cell activation [11]. However, evidence of virus-like particles (VLP) following DNA vaccination with SPDv full structural polyprotein has still to be demonstrated. Lastly, DNA vaccines do not need to generate a measurable primary antibody response to generate a protective secondary antibody response [45], and the results from the current study also do not exclude the possibility of other types of protective responses involving non-neutralising antibodies, e.g., complement activation or ADCC in vaccinated fish [46,47]. Based on non-detection of a measurable neutralising antibody response in the ppG-vaccinated fish group, our results suggest that T cell cytotoxicity may play an important role in ppG protection against SPDv1, as suggested for other fish species/DNA vaccine models [48].

Unfortunately, due to technical issues with the assay, we did not have sufficient material to follow antibody response in individual fish throughout the infection period. We were, however, able to link antibody levels in individual fish just prior to infection to terminal antibodies levels and to their viremia dynamics. No significant differences were observed between antibody levels in vaccinated fish pre- and post-infection (virus was detected in neither), potentially indicating no antibody induction during the challenge and no/minimum role for neutralising activity in the observed vaccine protection. It is possible that an early adaptive (or residual) antibody response in the vaccinated group resulted in limited replication of the virus, curtailing the vaccinated fish antibody response and keeping it below levels detectable by the assays used. In terms of strengths of the current approach, when antibody levels in pre- and post-infected fish injected with the empty vector were analysed, no differences were found when the group was analysed as a whole, but a significant difference pre-*p*-value-adjustment was found between pre- and post-infection antibody levels in fish that had early viremia, demonstrating that they mounted an antibody response to the virus within the timeframe of the experiment. Due to their delayed viremia, the “late viremia” group of fish may not have had time to develop a measurable antibody response in the interval to experiment termination. Salmon infected with SPDv through shedder cohabitation have been shown to develop neutralising antibody titres from 2 to 3 weeks post-exposure [14,35], but titres are variable [11,25]. High variation observed between individual fish in terms of antibody development in previous studies could, using this approach be interpreted more robustly through knowledge of their viremia dynamics and peak viral titres. Similarly, other early immune parameters could be linked to disease outcome or to vaccine efficacy in individual fish.

### 4.4. Plasmid Decay at the Site of Injection

The speed of decay of DNA at the site of injection was higher for the animals that had received the vaccine ppG compared to those that had received the 3.1H empty plasmid. This could reflect a potent cytotoxic reaction to transfected cells at the site of injection that express the SPDv proteins. Faster decay in levels of antigen-expressing plasmids has been documented in the past [49].

### 4.5. Immersion SPDv Challenge

Experimental infection by immersion challenge was used previously in freshwater where salmon fry (<2 g) were immersed in 5.10^4^ SAV3 (SPDv subtype 3) for 2 h [50] or in 0.3–3.10^5^ SAV1/5 (SPDv subtypes 1/5) for 4 h [51]. The present work is the first successful SPDv experimental infection by immersion of Atlantic salmon >30 g in seawater with cell-culture-produced virus. SPDv experimental infection using natural routes (immersion or cohabitation) induce very low mortality, and the success of infection is generally evaluated by histopathological examination, making the vaccine efficacy tests difficult and time-consuming [52]. High mortality rates can be obtained by injecting large doses of virus intramuscularly [53] but this procedure is not representative of the natural infection situation. A viremia based vaccine efficacy correlate may facilitate use of the latter natural infection route.

### 4.6. Early Type I Interferon and Later Vaccinal Protection

IFN is a key molecule linking innate and adaptive immunity to viruses [54]. Mice lacking functional IFN-α/β receptors were unable to generate influenza-specific antibodies in normal quantities [55], while co-administration of IFN and influenza vaccine increased specific antibody titres in mice when compared to vaccination alone [56]. IFN administered orally to mice enhanced the humoral or cellular adaptive immunity against Vaccinia Vaccine [57], and IFN increased antigen-specific CD8^+^T cells expansion and proliferation [58]. In fish, the importance of IFN induction for DNA vaccine efficacy is indirectly illustrated by the simultaneous observation of an early DNA vaccine-induced IFN response and high level of later specific protection within DNA vaccinated experimental fish groups [7]. In addition, certain DNA vaccines against fish viruses give a good level of protection only when adjuvanted with IFN-expressing plasmids [21,44,59].

The individual sequential monitoring allowed us to evaluate more robustly the potential importance of the early induction of type I IFN for the later adaptive protective ability of the DNA vaccine. We can see that in our experiments the DNA vaccine induced a type I IFN response at the site of injection and systemically, and this was also associated with reduced viremia in infected fish in a second experiment. On some individuals, we followed the level of *mx* expression, a well-characterised ISG, in the blood cells just after vaccination, as well as the viremia kinetics in the same fish after infection (Figure 6). We can clearly see that the absence of viremia is associated with a strong induction of type I IFN. The protection in the ppG group was total, and we therefore were not able to investigate correlations, if any, between the level of *mx* gene expression in blood cells and the level of reduction in viremia. The present work represents to date the most direct illustration of the association of an early IFN response with a later specific protection. Nevertheless, a clear demonstration remains to be established, preferably by the development of fish lines unable to respond to IFN type 1, similar to those developed for mice.

The chemokine gene γ*IP* and the T cell marker gene *cd8* showed changes in expression at the site of vaccine injection and systemically in the kidney over early time points post-vaccination. γ*IP* is generally thought to be produced by cells in response to IFNy stimulation, and as such is often considered a marker for IFNy induction. Following viral infection in mammals, IFNy is produced early and transiently by activated NK cells (though other lymphoid cells also produce the cytokine), and afterwards predominantly by CD4^+^ and CD8^+^ T cells once these have been activated by exposure to antigen [60]. IFNy activates macrophages to classical state and enhances antigen processing and presentation in macrophages and dendritic cells. While γ*IP* itself attracts leukocytes, macrophages, T cells and dendritic cells to sites of infection, it is also known to play a role in T cell development [61,62].

There was an initial decrease in *γIP* transcripts in ppG-vaccinated, and in *cd8* in ppG-vaccinated and empty-vector-injected fish kidney at 3 dpv, possibly indicating a migration of NK and CD8^+^ T cells from systemic lymphoid organs to the site of injection. Elevated levels of *γIP* transcripts were found in ppG-vaccinated fish at the site of injection at 3 dpv, followed by significantly increased expression of both *γIP* and *cd8* genes at 7 and 11 dpv. It has been suggested that the initial increase in *γIP* expression at the site of DNA vaccination in salmon at 7 dpv may be due to activated NK cells rather than activated CD4^+^/CD8^+^ cells, since the time to develop an antigen-specific T cell response is longer in cold-water fish [63]. The expression of *γIP* by NK cells may have promoted subsequent migration of CD8^+^ cells to the site of injection, resulting in the observed increase in *cd8* transcripts and the possible contribution of activated CD8^+^ cells to *γIP* production at 11 dpv (reference [64] found activated cytotoxic T cells at 10–11 dpv in VHSv vaccinated rainbow trout at 15 °C). The results indicate a role for cytotoxic T cells in the immune response to SPDv elicited by the ppG vaccine. Sobhkhez et al. [63] also concluded that the SAV3 (SPDv subtype 3) DNA vaccine may generate a strong T cell response, though the role in T cell-dependent antibody response or in T cell cytotoxicity needs to be confirmed by targeted studies. Though *γIP*, as mentioned, is often used as a marker for IFNy, the promoter of *γIP* in mammals and fish contains an IFNα/β ISRE sequence [65,66], and IFNα/β are reported to be potent inducers of the gene in the former model. It is therefore difficult to state conclusively that upregulation of *γIP* reflects IFNγ induction following SPDv DNA vaccination, but it is likely, based on the finding of IFNy involvement in other DNA vaccine models.

*γIP* and *mx* were also found upregulated in kidney tissue at 7 and 11 dpv in vaccine- but not in empty-vector-administered groups. It has previously been shown that DNA vaccine molecules can be transported to systemic locations following i.m. injection [67], potentially as naked plasmids in blood or following uptake in mobile APCs. This may account for the observed induction of interferon reporter genes in the kidney.

As discussed in previous studies, the DNA vaccine vector itself may induce an immune response through PPRs, e.g., CpGs associated with the plasmid backbone stimulating cells through TLRs, or double-stranded DNA structures signalling through cGAS-STING, IkB kinase (IKK) and TANK-binding kinase 1 (TBK1) [68,69]. In the current study, an increase in immune gene transcript expression/cell migration was observed in empty vector injected fish compared to PBS. However, a much greater upregulation of *mx*, *γIP*, and *cd8* was observed at the site of injection in vaccinated fish (Figure 2). This indicates that the viral protein itself, through its sequence, gene expression and/or presentation in cells up-taking the plasmid, is responsible for generating an immune response specific to the vaccine, including a strong IFN type 1 response, and is also responsible for longer-term protection as observed from results of the vaccine challenge. The results support in general those of [63]. While they observed significantly higher expression levels of *ifnγ* and IFN*γ*-induced genes (including *γIP*), as well as immune cell markers (including *cd8*), in the polyprotein vaccinated fish, they observed a similar increase in *ifn* type 1-induced gene transcripts following vaccination with both the empty vector and an SPDv subtype 3 polyprotein vaccine (pSPDV). They concluded that the pSPDv vaccine (as opposed to empty vector) generates a pronounced IFN*γ* stimulatory profile but, unlike the results of the current study (and as also seen for other fish virus DNA vaccines such as IHNv), does not specifically generate an IFN type I response). IFN type I and IFNy have both been reported to correlate with protection following DNA vaccination in a number of animal species and against a number of different viral diseases [70,71,72]. Zhu et al. [73] found in mice that NK maturation and activation following DNA vaccination was dependent on both IFN type 1 and the antigen expressed by the vaccine.

CD83 is a marker of dendritic cells in mammals and in fish [74,75]. Its expression was found upregulated at the site of injection in vaccinated fish at 7 dpv, again possibly due to the attraction of APCs to the site of injection through *γIP*. *il1b* transcripts, known to be produced by activated macrophages, were also observed to be significantly upregulated pre-*p*-value-adjustment at the site of injection only in fish injected with the ppG vaccine and only at 3 dpv, though a lower induction was also seen with the empty vector at 3 dpv. This upregulation agrees with previous studies where the *il1b* gene was induced at highest levels in vaccinated groups followed by empty vector at 3 dpv, followed by a lower and similar induction in both groups at 5 dpv [48]. Embregts et al. [48] suggested that a local proinflammatory response was generated by a combination of damage due to injection, the plasmid backbone and the vaccine target itself and is responsible for the recruitment of leukocytes to the site of injection. In relation to IL1B, its role in fish DNA vaccination requires further investigation, as numerous studies in mammals have demonstrated that IFNα/β, and indeed IFNγ, which are seen strongly upregulated in fish DNA vaccine trials including the current study, inhibit the cleavage of IL1B to its active form [76].

## 5. Conclusions

The advantages of sequential sampling and analysis from individual fish in understanding infection kinetics and host response are highlighted. This methodology allowed the observation of distinct differences in viremia kinetics between groups of fish in the same treatment, and the impact of this on subsequent antibody measurements at fixed sampling points. It is true that lethal sampling provides information on tissue-specific histopathological and immune responses. However, blood biomarkers for tissue-specific pathology [77] and immune response [78,79] in fish are continually improving. In combination with the sampling approach proposed here, we believe that significant improvements can be made in the understanding of disease dynamics at the population level, and the bases for differences in disease outcome, vaccine efficacy (protection correlates), and other biological phenotypes.

## Figures and Tables

**Table 1:** Histopathology scoring ladder used in this study. From Graham et al., 2011.

Tissue	Score	Description
Heart	0	Normal appearance
	1	Focal myocardial degeneration ± inflammation (<7 fibres affected)
	2	Focal myocardial degeneration ± inflammation (<15% of heart affected)
	3	Multifocal myocardial degeneration ± inflammation (>15 and <50% of heart affected)
	4	Severe diffuse myocardial degeneration ± inflammation (<50% of heart affected)
	R	Repair
Red and white skeletal muscle	0	Normal appearance
	1	Focal myocytic degeneration ± inflammation
	2	Multifocal myocytic degeneration ± inflammation
	3	Severe diffuse myocytic degeneration ± inflammation
	R	Repair

**Table 2:** Statistical analysis of the qPCR gene expression data from the Exp 1. Values in bold are *p*-values indicating significant differences in expression between indicated groups before adjustment for multiple comparisons, and * indicates adjusted *p*-value giving significance (*p* < 0.00119). B–H: Benjamini–Hochberg method (Benjamini and Hochberg, 1995) for adjusting *p*-values to accommodate type 1 errors following multiple t-tests, using a false discovery rate (FDR) of 0.05. dpv: days post-vaccination, PBS: PBS injected control fish, 3.1H: empty vector (pcDNA3.1-Hyg), ppG: DNA vaccine (pcDNA3.1-Hyg vector containing SPDv subtype 1 structural protein sequence).

		Muscle (Site of Injection)						Head Kidney						Blood Cells					
dpv	gene	Uncorrected *p*-Value			Corrected *p*-Value			Uncorrected *p*-Value			Corrected *p*-Value			Uncorrected *p* Value			Corrected *p*-Value		
		PBS v 3.1H	PBS v ppG	3.1H v ppG	PBS v 3.1H	PBS v ppG	3.1H v ppG	PBS v 3.1H	PBS v ppG	3.1H v ppG	PBS v 3.1H	PBS v ppG	3.1H v ppG	PBS v 3.1H	PBS v ppG	3.1H v ppG	PBS v 3.1H	PBS v ppG	3.1H v ppG
3	*mx*	0.8435	0.8401	0.9774	ns	ns	ns	0.2363	0.8740	**0.0479**	ns	ns	ns						
	*gIP*	0.2984	0.2016	0.1671	ns	ns	ns	0.3184	**0.0491**	0.1077	ns	ns	ns						
	*cd8*	0.4611	0.3760	0.9204	ns	ns	ns	**0.0046**	**0.0051**	0.8038	ns	ns	ns						
	*cd83*	0.5908	0.8532	0.6386	ns	ns	ns												
	*il1b*	0.1822	**0.0305**	0.6475	ns	ns	ns												
7	*mx*	**0.0131**	**0.0001**	**0.0002**	ns	*	*	0.5843	**0.0010**	**0.0011**	ns	*	*	0.0810	**0.0149**	**0.0194**	ns	ns	ns
	*gIP*	0.3994	**0.0043**	**0.0086**	ns	ns	ns	0.2039	0.7465	**0.0071**	ns	ns	ns						
	*cd8*	0.5121	**0.0017**	**0.0007**	ns	ns	*	0.2387	0.6095	0.7348	ns	ns	ns						
	*cd83*	**0.0248**	**0.0219**	**0.0100**	ns	ns	ns												
	*il1b*	0.3671	0.6218	0.1698	ns	ns	ns												
11	*mx*	0.2075	**0.0048**	**0.0050**	ns	ns	ns	0.6453	**0.0010**	**0.0011**	ns	*	*	0.1288	**0.0180**	**0.0130**	ns	ns	ns
	*gIP*	0.3259	**0.0439**	0.0534	ns	ns	ns	0.3677	**0.0297**	**0.0172**	ns	ns	ns						
	*cd8*	0.7784	0.1697	0.1791	ns	ns	ns	0.1708	0.2485	0.7811	ns	ns	ns						
	*cd83*	0.3940	0.8094	0.2950	ns	ns	ns												
	*il1b*	0.9824	0.8759	0.8680	ns	ns	ns												
46	*mx*	0.4706	0.5430	0.1777	ns	ns	ns	0.4432	0.1412	0.4686	ns	ns	ns						
	*gIP*	0.5673	0.2851	0.1196	ns	ns	ns	0.2002	0.1227	0.3344	ns	ns	ns						
	*cd8*	0.2869	0.9520	0.0669	ns	ns	ns	0.7191	0.7936	0.4058	ns	ns	ns						
60	*mx*							0.3101	0.2542	0.7686	ns	ns	ns						
	*cd8*							0.5566	0.9337	0.7346	ns	ns	ns						

## Data Availability

Data are available in a publicly accessible repository. The data presented in this study are openly available in Collet, Bertrand (2021), “DNA_vaccine_SPDv_Salmon”, Mendeley Data, V1, doi:10.17632/3nff493kdd.1 http://dx.doi.org/10.17632/3nff493kdd.1 under CC BY NC 3.0 license.
